# Bacterial Community Interactions During Chronic Respiratory Disease

**DOI:** 10.3389/fcimb.2020.00213

**Published:** 2020-05-14

**Authors:** Allison L. Welp, Jennifer M. Bomberger

**Affiliations:** ^1^Department of Microbiology and Molecular Genetics, University of Pittsburgh, Pittsburgh, PA, United States; ^2^Graduate Program in Microbiology and Immunology, University of Pittsburgh, Pittsburgh, PA, United States

**Keywords:** polymicrobial, biofilm, respiratory tract, pulmonary, antibiotic resistance

## Abstract

Chronic respiratory diseases including chronic rhinosinusitis, otitis media, asthma, cystic fibrosis, non-CF bronchiectasis, and chronic obstructive pulmonary disease are a major public health burden. Patients suffering from chronic respiratory disease are prone to persistent, debilitating respiratory infections due to the decreased ability to clear pathogens from the respiratory tract. Such infections often develop into chronic, life-long complications that are difficult to treat with antibiotics due to the formation of recalcitrant biofilms. The microbial communities present in the upper and lower respiratory tracts change as these respiratory diseases progress, often becoming less diverse and dysbiotic, correlating with worsening patient morbidity. Those with chronic respiratory disease are commonly infected with a shared group of respiratory pathogens including *Haemophilus influenzae, Streptococcus pneumoniae, Staphylococcus aureus, Pseudomonas aeruginosa*, and *Moraxella catarrhalis*, among others. In order to understand the microbial landscape of the respiratory tract during chronic disease, we review the known inter-species interactions among these organisms and other common respiratory flora. We consider both the balance between cooperative and competitive interactions in relation to microbial community structure. By reviewing the major causes of chronic respiratory disease, we identify common features across disease states and signals that might contribute to community shifts. As microbiome shifts have been associated with respiratory disease progression, worsening morbidity, and increased mortality, these underlying community interactions likely have an impact on respiratory disease state.

## Introduction

Rates of chronic respiratory disease (CRD) have increased with time and represent a major global health burden, comprising the fourth greatest cause of death (Heron, 2017). Chronic diseases are constant, persistent health issues causing significant morbidity and mortality. Individuals with CRD often have underlying defects in airway clearance, resulting in chronic respiratory infections. Chronic infections also involve the formation of biofilms, recalcitrant microbial communities that are able to persist, resisting immune clearance, and antibiotic killing.

Throughout the upper and lower human respiratory tract, niche-specific microbial communities colonize the entire length of the airways. Many of these organisms are found in complex microbial communities known as biofilms, which are encapsulated in a polysaccharide matrix. The upper respiratory tract (URT) contains the highest density of bacteria in the respiratory tract, but many of the healthy normal flora are highly shared between anatomical sites. The superior regions of the respiratory tract, the sinonasal and nasal cavities, share a similar microbiome, including *Corynebacterium* spp. *, Staphylococcus* spp., and *Cutibacterium acnes* (Ramakrishnan et al., 2016; Man et al., 2017; Paramasivan et al., 2020). Beginning in the nasal cavity and extending to the oropharynx, other bacteria are found including *Moraxella catarrhalis, Haemophilus* spp., and *Streptococcus* spp. Most of the species found in the lower respiratory tract (LRT) are common oral flora, suggesting that microaspiration of mouth contents results in more colonization than anything inhaled from the air (Dickson and Huffnagle, 2015). Such organisms include *Prevotella, Veillonella*, and *Rothia* spp., in addition to *Streptococcus* spp. (Man et al., 2017). However, these studies inherently have potential limitations due to the nature of sample collection. In sampling the LRT, a bronchoscope must pass through the URT, providing a source of potential contamination. Many studies have concluded the community signatures of the LRT are likely not due to sample contamination through the collection of matched oral wash samples, repeated sampling of the same site, and through various routes of insertion (oral versus nasal) (Dickson et al., 2014; Bassis et al., 2015; Dickson and Huffnagle, 2015). Therefore, proper controls are essential when studying these distal sites.

The importance of the human microbiome to overall health is well-appreciated, and many studies have demonstrated that dysbiosis of the normal microbial communities in any particular anatomical site is strongly associated with disease progression. A healthy microbiome contains a diverse community of organisms. Such communities are markedly more stable and resistant to blooms of pathogenic species. Commensal bacteria maintain community homeostasis by secreting antimicrobial peptides and other molecules to suppress the growth of pathogens (Gallo, 2015). However, in dysbiotic communities associated with chronic disease, the diversity is markedly lower, often dominated by a few pathogenic species with diminished normal healthy flora. Notably, dysbiotic communities are more susceptible to inflammation and immune activation. The acquisition or presence of certain pathogenic species might drive spatial organization, community composition, and functional behaviors. Commensal flora usually limits the growth of pathogens, but when these communities are compromised, the proliferation of pathogenic species during dysbiosis may trigger inflammation and lead to chronic inflammation and disease progression (Lawley et al., 2009).

Early in life, a number of environmental factors likely shapes the developing microbiome and potentially dictates future respiratory health. Notably, by 1–2 months of age, infants' microbiomes diversify and develop a unique microbial signature in their upper airways, which seem to correlate with predisposition to respiratory infections (Bosch et al., 2016). Infants dominated by *Corynebacterium* spp., *Staphylococcus* spp., or *Alloiococcus* spp., had a lower predisposition to respiratory illnesses, whereas infants with *Streptococcus, Moraxella*, or *Haemophilus* dominated microbiomes had higher rates of respiratory illness and predisposition to asthma, likely due to the inflammatory potential of these organisms (Teo et al., 2015; Bosch et al., 2016). These outcomes depended largely on exposure to antibiotics as an infant, the presence of siblings, and season (Teo et al., 2015).

In patients with chronic respiratory disease, a number of factors contribute to an altered respiratory microbiome. The production of excess mucus in diseases such as cystic fibrosis and chronic rhinosinusitis provide additional nutrient sources as well as pockets of decreased oxygen availability, allowing for different species to colonize these new niches. Additionally, decreased mucociliary clearance resulting from the pathogenesis of diseases such as bronchiectasis and cystic fibrosis result in decreased microbial clearance and elimination, allowing environmental microbes increased access to these sites. Disease exacerbations and the acute worsening of respiratory symptoms result in many interventions, including antibiotic therapy and hospitalizations, that have the potential to disrupt the microbiome. Respiratory symptoms themselves also allow for microbial migration and elimination including cough, hyperventilation (microaspiration), and bronchoconstriction, resulting in altered oxygen availability, temperature, and pH (Dickson and Huffnagle, 2015). Approximately 75% of individuals experiencing chronic lower respiratory tract disease also develop esophageal reflux and dysfunction, allowing for microbial migration from the digestive tract into the respiratory tract (Dickson and Huffnagle, 2015).

Although many of these respiratory symptoms are shared among various chronic respiratory diseases, each is uniquely classified by the anatomical location of inflammation and infection. Different regions of the respiratory tract differ in their physiochemical properties, including temperature, pH, and nutrient availability, like oxygen (Dickson et al., 2016). These factors likely impact the microbial communities present and influence disease progression.

In the following review, we discuss the underlying pathophysiology of common chronic respiratory diseases, the microbial communities present in the diseased respiratory tract, and common pathogens and exacerbations that impact lung function. In relation to these communities, we discuss both cooperative and competitive species interactions that occur and how these likely impact disease state and the overall microbial communities. Although much more work is needed to truly understand daily microbiome changes *in vivo*, these interactions have the potential to underly many CRD manifestations.

## The Microbiology of Chronic Respiratory Diseases

### Chronic Rhinosinusitis

Chronic rhinosinusitis (CRS), the chronic inflammation of the nose and paranasal sinuses, affects ~31 million people in the US alone (Bose et al., 2016) (12.5% of US adults; 11% of European adults) with an economic burden of over 60 billion dollars (Caulley et al., 2015). CRS results in impaired mucociliary clearance and poor drainage of the sinuses, resulting in mucus accumulation, but this disease has been hypothesized to develop in response to microbial dysbiosis and associated inflammation (Lam et al., 2015; Jervis Bardy and Psaltis, 2016; Hoggard et al., 2017; Wagner Mackenzie et al., 2017; Copeland et al., 2018). The CRS sinuses are distinctly less diverse than healthy controls, however whether low diversity and dysbiosis is the cause of CRS or a consequence of the disease is still unclear (De Boeck et al., 2019). Regardless, the CRS microbiome is enriched in *Cutibacterium acnes* and *Corynebacterium* spp., with a known enrichment of *Corynebacterium tuberculstearicum* in CRS patients (Abreu et al., 2012). CRS patients also harbor increased number of pathogens including *Staphylococcus aureus*, coagulase-negative *Staphylococcus* spp., *Streptococcus pneumoniae*, and *Haemophilus influenzae* in their sinuses. Notably, the presence of *S. aureus* or *H. influenzae* correlates with the development of nasal polyps, inflammatory lesions which contribute to worsening disease morbidity (Bose et al., 2016; Chalermwatanachai et al., 2018). Patients with CRS often experience disease exacerbations, characterized by a sudden worsening of respiratory symptoms. These exacerbations have been associated with acquisition of other bacterial pathogens, including *Pseudomonas aeruginosa, Proteus mirabilis*, and *Klebsiella pneumoniae* (Bose et al., 2016).

The complex environment of the inflamed sinuses of CRS patients, consisting of an influx of inflammatory immune cells, effector molecules, and cationic antimicrobial peptides, likely have a direct impact on bacterial gene expression and resulting microbial community structure. The presence of certain species also likely has an impact on the resulting community structure and virulence of CRS pathogens. Specifically, the presence of large numbers of *Corynebacterium* spp. might drive *S. aureus* to be less virulent and more benign, whereas the presence of certain Proteobacteria, such as *E. coli*, might drive *S. aureus* to exhibit more pathogenic behavior (Ramakrishnan et al., 2015, 2016). Interspecies interactions might also contribute to worsening disease pathology. For example, as *H. influenzae* is associated with occurrence of nasal polyps in CRS patients, it is thought that *H. influenzae* might actually initiate and drive inflammation and impact the development of nasal polyposis (Chalermwatanachai et al., 2018). These associations might have important implications for treatment of CRS, including early elimination of *H. influenzae* or *S. aureus* to prevent the development of nasal polyps.

### Otitis Media

Otitis media, inflammation of the middle ear, is the most frequently diagnosed illness in children under 15 and the primary cause for emergency room visits (Cassell et al., 1994). Approximately 80% of children will experience OM by age 3, affecting between 330 and 700 million people worldwide (Teele et al., 1989; Monasta et al., 2012; Mittal et al., 2015). Acute OM can progress to chronic suppurative OM (CSOM), which is associated with ear drum perforation and purulent discharge (Acuin, 2007). There are around 31 million new cases of CSOM per year, with 22.6% of cases in children under 5 years old (Monasta et al., 2012). Pathogens cause infection and subsequent disease by gaining access to the middle ear through the Eustachian tube. As early as six months of age, many children are already colonized by bacteria known to cause OM, including *Moraxella catarrhalis, Streptococcus pneumoniae*, and *H. influenzae* (Bakaletz, 2010; Qureishi et al., 2014). Of these species, *S. pneumoniae* is the most virulent and inflammatory, causing a highly symptomatic disease, while *H. influenzae* and *M. catarrhalis* are more likely silent, chronic infections (Bakaletz, 2010). However, *P. aeruginosa* and *S. aureus* are the most common pathogens found in CSOM cases (Mittal et al., 2015). An important aspect of OM pathophysiology is the presence of bacterial biofilms in the ear, allowing for chronic, persistent infection. The presence of biofilms in the middle ear exudate has been confirmed by microscopy (Kaya et al., 2013; Gu et al., 2014). Particularly, all three major pathogens (*H. influenzae, S. pneumoniae*, and *M. catarrhalis*) have been found together in polymicrobial biofilms (Kaya et al., 2013).

Continuing to understand how OM pathogens contribute to disease pathology is important to understand treatments. Colonization is likely an important step in dictating the course of disease progression. Natural competition likely occurs in the middle ear and Eustachian tube to impact pathogenesis and microbial communities. For example, the presence of *Corynebacterium* spp. is often inversely correlated with *S. pneumoniae* and *S. aureus* colonization (Kumpitsch et al., 2019). Additionally, children with recurrent OM who were vaccinated with the 7-valent pneumococcal vaccine had an increased incidence of *S. aureus* and *H. influenzae* disease and colonization, also suggesting a competitive relationship of *S. pneumoniae* with *S. aureus* and *H. influenzae* (Biesbroek et al., 2014).

### Asthma

Over 300 million people suffer from asthma, and this number continues to climb (Nunes et al., 2017). This chronic inflammatory disease results in narrowing of the airways and reduction in airflow. Asthma is the most frequent chronic disease among pediatric patients (Boutin et al., 2017). During an asthma attack, the lining of the bronchial tubes become inflamed, narrowing the airways and resulting in widespread reduction in airflow. Many factors have been associated with the development of asthma including geographical location, viral infection, and antibiotic exposure (Ni et al., 2019). There is evidence suggesting that infection with *Chlamydia pneumoniae* or *Mycoplasma pneumoniae* may precede the development of asthma (Hahn et al., 1991). Other studies have suggested that exposure to *S. aureus* and its associated enterotoxins early in life might also be a risk factor for development of asthma (Redinbo, 2014). *S. aureus* enterotoxins function as superantigens, which might result in damage of the lower and upper respiratory tract and eosinophil accumulation. To support this hypothesis, antibodies specific to *S. aureus* enterotoxins are more commonly found in asthma patients as compared to healthy controls (Redinbo, 2014).

The respiratory microbiome also has important implications for treatment response. Previously, patients who failed to respond to the inhaled corticosteroid fluticasone had more dysbiotic respiratory microbiomes, whereas those who responded favorably had microbiomes more similar to healthy controls (Durack et al., 2017). As compared to healthy individuals, individuals with asthma have a respiratory microbiome enriched in *Haemophilus* spp. *, Neisseria* spp. *, Fusobacterium* spp., and *Porphyromonas* spp. (Durack et al., 2017). Specifically, infection with *Streptococcus pneumoniae* was associated with asthma exacerbations (Iikura et al., 2015). Long-term, chronic colonization of the airways may result in neutrophilic accumulation observed in a subset of asthma patients. In those with neutrophilic asthma, *M. catarrhalis* was most often associated with worsening disease, but *H. influenzae* and *Streptococcus* spp. often also dominated the airway microbiome (Green et al., 2014). Infection with these three species was associated with increased duration of disease, worse lung function (FEV_1_), and higher sputum neutrophil counts and IL-8 concentration (Green et al., 2014).

### Cystic Fibrosis

The genetic disease cystic fibrosis (CF) results from mutation in the cystic fibrosis transmembrane conductance regulator (CFTR), an ion channel primarily responsible for regulating chloride and bicarbonate transport (Elborn, 2016). This defect alters the pH of airway surface liquid, decreases mucociliary clearance, and impairs innate immunity. Chronic respiratory infections are the leading cause of death in people with CF, and disease exacerbations are a major cause of pulmonary function decline in CF patients (Stenbit and Flume, 2011). Early in life, the LRT of CF patients are predominately colonized with oral bacteria including *Streptococcus* spp., *Prevotella* spp., and *Veillonella* spp. (Zemanick et al., 2017) The most common CF pathogens isolated from young CF patients include *S. aureus* and *H. influenzae*. However, acute infections with these pathogens transition into chronic respiratory infections with age. As patients age, their microbiome becomes less diverse and more dominated with one or a few CF pathogens including *P. aeruginosa, Burkholderia cepacia* complex, *Stenotrophomonas maltophilia, Achromobacter xylosoxidans*, or *S. aureus* (Coburn et al., 2015). This shift might be due to respiratory viral co-infections, as preceding viral infections are associated with *P. aeruginosa* acquisition, development of chronic *P. aeruginosa* infection, and increased *P. aeruginosa* antibodies (Petersen et al., 1981; Johansen and Høiby, 1992; Collinson et al., 1996).

Specific CF pathogens likely strongly shape the microbial communities present in the CF lung and contribute to the microbial shifts documented as patients age. Specifically, the presence of *P. aeruginosa* and *B. cepacia* complex, pathogens commonly isolated in adult CF patients, notably impact the composition and presence of other species (Stressmann et al., 2012; Zemanick et al., 2017). The presence of either one of these organisms is associated with lower odds of infection by methicillin-sensitive *S. aureus, S. maltophilia*, or *A. xylosoxidans* in the future (Granchelli et al., 2018). These two pathogens are thought to directly contribute to declining microbial diversity due to their ability to compete with other species found in the CF lung, both limiting the acquisition of other pathogens and dominating over pre-established microbial communities. *P. aeruginosa* and *B. cepacia* complex co-infections are also reported, and co-infection is associated with worsening disease severity and lung function in both mice and humans, as compared to mono-infection (Isles et al., 1984; Jacques et al., 1998; Bragonzi et al., 2012).

### Non-CF Bronchiectasis

Non-CF bronchiectasis comprises a disease characterized by abnormal widening of one or more airways, with extra mucus pooling in these airways (McShane et al., 2013). Bronchiectasis pathophysiology may be due to ciliary dyskinesia, autoimmune disease, immune deficiencies, hypersensitivities, TB, or, other infections. Approximately 350,000–500,000 adults have bronchiectasis in the US, and its incidence increases with age, especially in adults over the age of 60 (Chalmers and Elborn, 2015). People with bronchiectasis experience chronic cough, viscous sputum, frequent hospitalizations, and often one or more disease exacerbations per year. Exacerbations not only contribute to worsening morbidity, but also mortality, as patients who experience three or more exacerbations per year have double the mortality rate as those who have fewer (Chalmers et al., 2013). During exacerbation, bacterial density in the respiratory tract does not change, but microbial community shifts occur, including the increased prevalence of anaerobes in the LRT (Tunney et al., 2013). The most predominant species colonizing the LRT of bronchiectasis patients include *H. influenzae, S. pneumoniae, S. aureus, P. aeruginosa, M. catarrhalis, Prevotella*, and *Veillonella*. Over time, the lung microbiome of bronchiectasis patients seem to be largely dominated by either *P. aeruginosa* or *H. influenzae*, although these two organisms are rarely isolated together, suggesting there is natural competition among these species (Rogers et al., 2013; Woo et al., 2019). However, many studies have shown that patients infected with *P. aeruginosa* experience the most rapid decline in lung function (FEV1%) and have the highest hospital admission rates (Finch et al., 2015; McDonnell et al., 2015). In both CF and non-CF bronchiectasis, *P. aeruginosa* undergoes clonal selection and diversification over time, reflecting its ability to adapt to the diseased lung (Hilliam et al., 2017). These adaptations and mutations demonstrate the need for limited nutrients, biofilm formation, and competition with other organisms.

### Chronic Obstructive Pulmonary Disease (COPD)

One of the greatest contributors to chronic respiratory disease is chronic obstructive pulmonary disease (COPD). This disease alone is the third-leading cause of death worldwide, and over 65 million people suffer from moderate to severe COPD (Quaderi and Hurst, 2018). COPD results in the chronic obstruction of airflow (obstructive bronchitis), impairing normal breathing, and/or destruction or the lung parenchyma, often caused by exposure to noxious gases including cigarette smoke. Patients with COPD experience symptoms including chronic cough, mucociliary dysfunction, excess mucus production, and breathlessness. This environment favors colonization from a number of bacteria, including predominant species such as *H. influenzae, S. pneumoniae*, and *M. catarrhalis* (Beasley et al., 2012).

COPD patients are found to have significantly lower microbial community diversity in the LRT (Quaderi and Hurst, 2018). Many factors seem to influence this shift. Disease exacerbations and corticosteroid use have been associated with decreased diversity and enrichment of specific species (Wang et al., 2016). It has been observed that, as the disease progresses and airways become more obstructed, the prevalence of *P. aeruginosa, H. influenze*, and other Gram-negative pathogens increase (Soler et al., 1999; Murphy et al., 2008; Engler et al., 2012). Disease exacerbations are most commonly associated with the presence of *H. influenzae* in the airways, but are also associated with acquisition of new strains of *P. aeruginosa, H. influenzae*, or *M. catarrhalis* (Soler et al., 1999; Mammen and Sethi, 2016). Notably, in microbiome analyses of COPD patients, those who were colonized with *H. influenzae* had many negative correlations with species found in healthy individuals (Wang et al., 2016). Therefore, the presence of *H. influenzae* might directly impact the composition of the lung microbiome. Finally, patients with low community diversity also had higher levels of serum IL-8, suggesting that the microbiome might drive inflammation and directly impact disease severity (Wang et al., 2016). By understanding the microbial interactions which lead to microbial community changes and decreased diversity, we can better understand how the microbiome contributes to disease progression and how to prevent these shifts.

## Microbial Community Interactions

Even the earliest reports of microorganisms have noted that species are rarely found in isolation. Very few body sites in humans are thought to be truly sterile. With sequencing advances, we continue to discover that healthy humans harbor microbial communities in areas previously assumed to be sterile, including the lower respiratory tract where many species are found to co-exist (Harrison, 2007; Dickson and Huffnagle, 2015). Unfortunately, our understanding of bacterial pathogenesis is often restricted to species in mono-culture. How community members interact in combination is often different and difficult to predict by studying individual species. When pathogenesis is studied with the addition of other species or in polymicrobial environments, bacterial interactions, and associated disease severity sometimes changes (Duan et al., 2003; Sibley et al., 2008; Korgaonkar et al., 2013).

In the airways, bacteria are localized in biofilm communities to persist and resist clearing from the host. Biofilms have been directly detected in CRD including CRS (Cryer et al., 2004; Sanclement et al., 2005), CF (Bjarnsholt et al., 2013; Hoiby et al., 2017), OM (Hall-Stoodley et al., 2006; Bakaletz, 2012; Gu et al., 2014), and bronchiectasis (Marsh et al., 2015) and have been implicated in other diseases including COPD (Murphy et al., 2005; Pang et al., 2008). The spatial organization of these communities favor the close proximity of related cells due to growth and clonal expansion. This arrangement limits the diffusion of extracellular products away from a population of related cells to restrict access from competitors and reduce cheating. However, this dense environment also allows for close contact with competing species and renders a population vulnerable to direct antagonistic attacks.

Bacteria have evolved multiple mechanisms to interact with neighboring cells to acquire the nutrients necessary to survive, resist clearing from the host during infection, and persist over time. Such interactions have differing effects on other species, broadly resulting in either cooperative behaviors or competitive interactions. While many of these interactions rely on the use and exploitation of the greater nutritional environment and production of extracellular products, some of these interactions are more targeted, with specific cross-talk mediated by direct contact.

There have been a wealth of studies describing the pathogenesis and coordination of these behaviors *in vitro*, as well as a number of studies detailing known clinical associations and microbial community shifts during CRD. However, the role of these interactions during human disease is only speculative. Here, we will review known microbial interactions in light of observed disease shifts in an effort to identify connections and enhance our understanding of chronic respiratory disease progression.

### Cooperative Behaviors

**Figure 1:**
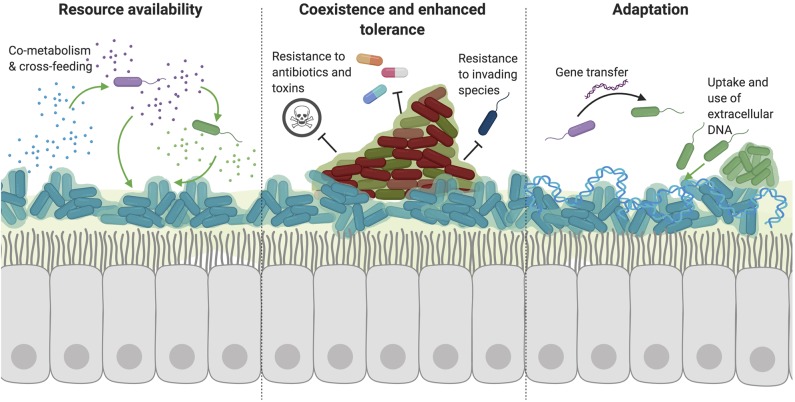
Cooperative interactions favor the persistence of multiple species. A number of cooperative interactions have been described among species, which can broadly be broken down into three main categories. (1) Bacteria benefit from the resources released or metabolized by other species. Through mechanisms such as co-metabolism and cross-feeding, species can utilize complex nutrients that they may be unable to metabolize or access themselves. Additionally, the secretion of public goods from one species (such as siderophores, enzymes, or signaling molecules) also benefits neighboring bacteria by releasing resources into the extracellular environment. (2) The coexistence of multiple species of bacteria in a polymicrobial biofilm also allows the sharing of resources and cross-species signaling due to the close proximity of these species. Importantly, certain species can protect and shield other neighboring species in a biofilm through enhanced tolerance to antibiotics, immune radicals, and other toxic small molecules. (3) Interactions with other species also result in adaptation to improve survival in the host environment. While many organisms might evolve to evade toxic effects from competitive species, adaptation also occurs through horizontal gene transfer and uptake of exogenous bacterial DNA from the environment. The presence of this DNA alone can also aid in biofilm formation through incorporation into the extracellular biofilm matrix.

Although bacteria often compete for space and resources to persist in the body, a number of cooperative interactions have been described between species. Such interactions often positively impact both species and favor the good of the group rather than an individual cell (Figure 1). Many of these cooperative interactions have been described in common respiratory commensals and CRD pathogens.

#### Cross-Feeding

Bacteria rely on specific nutrients to survive in the nutrient-restricted host environment of the respiratory tract (Armstrong, 2015). However, many nutrients are not readily available because they are sequestered by the host or require breakdown of larger molecules. Some species of bacteria are able to utilize and catabolize complex molecules to access needed nutrients. Other species can use the metabolites produced by these organisms as a source of energy or nutrient sources in a process called cross-feeding (Seth and Taga, 2014). Many carbohydrate sources are available in the respiratory tract and are implicated in polymicrobial community interactions, as recently reviewed by Armbruster et al. (2019), but one common example is mucus, predominately comprised of mucins. Mucin is one of the most abundant molecules in the respiratory tract and is produced in large quantities in CRD, including CF and COPD. These molecules are comprised of an amino acid backbone with oligosaccharide side chains. Terminal domains on the oligosaccharide chains form a highly cross-linked structure, making most of these nutrients inaccessible to bacteria (Flynn et al., 2016). Species including *P. aeruginosa* and *S. aureus* cannot efficiently use mucin as a sole carbon source (Flynn et al., 2016). However, the presence of other commensal species including *Rothia mucilaginosa, Streptococcus* spp., *Prevotella* spp., and Veillonella spp., cooperate to degrade mucin and liberate useable metabolites for other species (Flynn et al., 2016; Gao et al., 2018). These species, along with other anaerobes, posess glycoside hydrolase, sulfatase, sialidase, alpha-fucosidase, and endopeptidase activities (Bradshaw et al., 1994; Wright et al., 2000; Inui et al., 2015). The unique enzymatic activities of anaerobic species act synergistically to release fermentative metabolites, including amino acids, such as glutamate, and short-chain fatty acids, including proprionate, acetate, and butyrate (Bradshaw et al., 1994; Mirković et al., 2015).

Notably, these metabolites were found to be increased in adult CF patient sputum (Mirković et al., 2015). Therefore, anaerobic species which are found in high numbers in sputum from people with CRD, can support the growth of common pathogens including *P. aeruginosa, S. aureus*, and *Burkholderia cenocepacia* through the cooperative utilization and metabolism of mucins (Tunney et al., 2008; Flynn et al., 2016). Such behavior increases available resources and results in cooperation (Figure 1).

#### Public Goods

In addition to nutrients, bacteria can also produce extracellular products that benefit neighboring cells. Although an individual cell experiences an energetic cost in metabolism, the production is beneficial for the surrounding population (Zhao et al., 2019). The production of these products occurs when bacteria are in a dense biofilm community where many of the surrounding cells are related organisms. These processes are often coordinated by quorum sensing, where bacteria release signal molecules (autoinducers) as a measure of cell density. Once this signal reaches a certain concentration, a change in gene expression occurs (Miller and Bassler, 2001). In this way, an organism regulates the production of goods until it is present at a sufficient cell density to benefit from production. A number of extracellular bacterial products can be exploited as public goods including extracellular enzymes, surfactants, exopolysaccharides, virulence factors, and siderophores. For example, the *P. aeruginosa* polysaccharide Psl functions as a public good, as it is an important factor for attachment to surfaces and biofilm formation (Irie et al., 2017). Production of the Psl polysaccharide was found to benefit bacterial populations and has social benefits for *P. aeruginosa* strains that do not produce Psl (Hall-Stoodley et al., 2006). However, production of public goods is highly prone to exploitation by social cheating. In this way, “cheaters” benefit from the production of public goods but do not expend energy for production. Species, such as *P. aeruginosa*, limit cheating by coregulating the production of public goods (such as protease) with the production of virulence factors to limit the growth of other organisms, including pyocyanin, hydrogen cyanide, and rhamnolipid production (Smalley et al., 2015).

#### Inter-Species Signaling

Quorum sensing is also involved in inter-species signaling. Although known autoinducer molecules are quite diverse, one signal is highly conserved across many species. This signal, autoinducer-2 (AI-2), is produced by the LuxS synthase found in bacteria including *Haemophilus* spp., *Staphylococcus* spp., and *Streptococcus* spp. (Federle and Bassler, 2003). In *H. influenzae*, AI-2 is necessary for biofilm maturation, persistence, and chronic infection in a chinchilla model (Armbruster et al., 2009). However, AI-2 also has important roles in inter-species gene regulation and the formation of mixed-species biofilms. Specifically, *M. catarrhalis* displays increased biofilm formation and enhanced antibiotic tolerance in co-culture with *H. influenzae* (Armbruster et al., 2010). Although *M. catarrhalis* does not encode LuxS, it is able to detect the production of AI-2 by *H. influenzae* in order to promote biofilm, allowing *M. catarrhalis* to “eavesdrop” on nearby species. The addition of exogenous AI-2 precursor alone to mono-cultures of *M. catarrhalis* was sufficient to increase biofilm (Armbruster et al., 2010). Similar responses to AI-2 also occur in *P. aeruginosa*, which also lacks LuxS synthase. In response to AI-2, *P. aeruginosa* produces phenazines, elastase, rhamnolipids, flagellin, and other genes, likely altering its interactions with nearby microbes (Duan et al., 2003). AI-2 has been detected from CF patient sputum, suggesting that this molecule is especially important for bacterial pathogenesis *in vivo* (Duan et al., 2003).

#### Formation of Polymicrobial Biofilms

In the respiratory tract, pathogens encounter many other organisms which may alter the organization of bacterial communities. For example, changes in *P. aeruginosa* and *S. aureus* biofilm formation have been shown to result from co-infection with other organisms, notably respiratory viruses such as respiratory syncytial virus and rhinovirus (Hendricks et al., 2016; Kiedrowski et al., 2018). Bacterial species can also impact neighboring bacteria to increase biofilm formation, facilitating enhanced tolerance, and persistence of both species. These changes are sometimes due to altered bacterial signaling and metabolite production that occurs during polymicrobial infections. As described above, AI-2 production by *H. influenzae* enhances the biofilm formation of *M. catarrhalis* (Armbruster et al., 2010). Many other species also display increased biofilm formation during co-culture. Although many of the specific signals preceding this change are still unknown, a number of observations have been made. First, clinical isolates of *P. aeruginosa* and *Burkholderia cepacia* from CF patients were observed to form mixed species biofilms during co-culture (Tomlin et al., 2001). Other studies found that *P. aeruginosa* conditioned media enhanced the attachment of *B. cepacia* to epithelial cells, suggesting that *P. aeruginosa* signals to *B. cepacia* to enhance biofilm formation through an unknown mechanism (Saiman et al., 1990). Additionally, the presence of *P. aeruginosa* also enhanced subsequent biofilm formation of *Klebsiella pneumoniae* (Stewart et al., 1997).

Altered gene expression during co-culture in polymicrobial biofilms likely underlies most of these observed changes. Specifically, the relationship between *H. influenzae* and *S. pneumoniae* is well-documented. These two pathogens both reach higher cell densities during co-culture as either do alone (Margolis et al., 2010; Cope et al., 2011). Additionally, *H. influenzae* reaches a higher cell density when invading established populations of *S. aureus* or *S. pneumoniae* (Margolis et al., 2010). Transcription of the *H. influenzae* type IV pili occurred only in the presence of *S. pneumoniae*, as compared to monoculture. Expression of type IV pili was also confirmed in diseased tissue from CRS patients. The type IV pili is an essential factor for *H. influenzae* biofilm formation and might also mediate direct contact with other species during co-culture, so induction of type IV pili during co-culture is likely an important factor mediating colonization and persistence of *H. influenzae* during respiratory infection (Jurcisek et al., 2007).

Coaggregation of bacterial species may also impact the development of polymicrobial biofilms, as the association of genetically distinct bacteria has previously been shown to enhance bacterial adhesion preceding biofilm formation (Rickard et al., 2003). A few examples of coaggregation have been reported in the respiratory tract, as *M. catarrhalis* can coaggregate with *Streptococcus pyogenes*, enhancing the colonization and adherence of *S. pyogenes* to epithelial cells (Lafontaine et al., 2004). However, the role of coaggregation in the respiratory tract is still poorly understood (Humphreys et al., 2017).

#### Enhanced Tolerance

The presence of other microbes can confer higher antimicrobial resistance and enhanced protection from immune radicals to the entire microbial population through a variety of mechanisms (Figure 1). First, *P. aeruginosa* production of extrapolymeric substance (EPS), an essential component of the biofilm matrix, provides protection to other neighboring species (Billings et al., 2013; Periasamy et al., 2015). *P. aeruginosa* produces three different polysaccharides with various roles in biofilm formation—Psl, Pel, and alginate (Hentzer et al., 2001; Colvin et al., 2012). All three polysaccharides have been documented to confer antibiotic resistance to other bacteria. Non-mucoid strains of *P. aeruginosa* primarily used Pel or Psl as a primary matrix polysaccharide. Psl has been found to confer protection to *E. coli* and *S. aureus* to colistin and tobramycin, respectively (Billings et al., 2013). This effect is thought to be due to binding and sequestration of antibiotics to the matrix, limiting its contact with the bacterial cell surface. Although no effect on other species has been described, Pel and extracellular DNA (eDNA) have both been shown to enhance resistance to aminoglycosides, suggesting they might also protect other species from aminoglycoside toxicity (Khan et al., 2010; Colvin et al., 2011; Chiang et al., 2013). Mucoid strains of *P. aeruginosa* are commonly isolated during chronic infection, especially in CF (Bjarnsholt et al., 2009). The conversion of *P. aeruginosa* to a mucoid phenotype involves mutations resulting in the overexpression of alginate (Hentzer et al., 2001). The expression of alginate is known to confer enhanced antibiotic tolerance of *S. maltophilia* to tobramycin (Pompilio et al., 2015) and ciprofloxacin (Magalhães et al., 2017) as well as *S. aureus* and *Inquilinus limosus* resistance to ciprofloxacin (Magalhães et al., 2017). Alginate is thought to impact antibiotic resistance by slowing or preventing antibiotic penetration into the biofilm matrix (Gordon et al., 1988; Nichols et al., 1988). The expression of alginate was also increased when *P. aeruginosa* and *S. maltophilia* are grown in a polymicrobial biofilm, suggesting this mechanism is important in polymicrobial environments (Pompilio et al., 2015).

Antibiotic tolerance can also be conferred to other species by enzymatic activity, delivery of these enzymes, and horizontal gene transfer resulting in the acquisition of antimicrobial resistance genes. The production of β-lactamase, which functions to cleave the β-lactam ring of β-lactam antibiotics, by non-typeable *H. influenzae* (NTHi) is shown to protect both planktonic and biofilm-associated *S. pneumoniae* from amoxicillin (Weimer et al., 2011). However, β-lactamases can also confer inter-species protection through the delivery in bacterial outer-membrane vesicles (OMVs). *M. catarrhalis* is able to protect *S. pneumoniae* and *H. influenzae* from amoxicillin treatment through the transfer of β-lactamases in OMVs (Schaar et al., 2011). Delivery of β-lactamase in OMVs is likely important to prevent enzymatic degradation during transfer to other cells, as shown for *P. aeruginosa* and *S. aureus* (Ciofu et al., 2000; Bomberger et al., 2009; Lee et al., 2013).

Polymicrobial communities also protect neighboring species from the host immune system. The presence of bacteria can modulate both immune cell function and any toxic substances they release. *S. aureus* alpha toxin (AT) is able to inhibit human mononuclear cell antibacterial activity (Cohen et al., 2016). Specifically, AT impaired phagocytosis of *S. aureus, P. aeruginosa*, and *K. pneumoniae*. Therefore, *S. aureus* AT allows proliferation of other microbes by inhibiting immune cell function. Immune cells, including macrophages, release toxic metabolites as signaling molecules which are capable of killing pathogens. Nitric oxide (NO), one such free radical, is capable of inducing DNA damage and degrading iron sulfur clusters, inhibiting bacterial growth (Wink and Mitchell, 1998). However, *S. aureus* is quite resistant to NO due to its detoxification abilities (Grosser et al., 2018). In co-culture with *P. aeruginosa, S. aureus* detoxifies NO to promote the survival of CF clinical strains of *P. aeruginosa*, consistent with findings showing *S. aureus-P. aeruginosa* co-infection in CF patients (Hoffman et al., 2010; Limoli et al., 2016). Additionally, bacterial relationships are also shaped by the presence of other host proteins involved in the immune response. Calprotectin, a protein released from neutrophils or other phagocytes, sequesters essential nutrients including iron, zinc, and manganese away from bacteria in order to inhibit their growth (Zygiel et al., 2019). Iron-, zinc-, and manganese- limited environments in turn repress the production of anti-staphylococcal factors in *P. aeruginosa*, promoting increased levels of *P. aeruginosa* and *S. aureus* co-colonization *in vitro* and *in vivo* (Wakeman et al., 2016).

#### DNA Availability

Extracellular DNA (eDNA) is an integral component of the biofilm matrix (Whitchurch et al., 2002), but also serves many other purposes in microbial communities (Figure 1). The presence of DNA in the host environment results from a variety of processes including lysis of cells due to lytic bacteriophage, autolysis, cell death, and active secretion of DNA (Steinmoen et al., 2002; Jurcisek et al., 2017). Extracellular DNA is produced by organisms including, *P. aeruginosa, Streptococcus* spp., *Staphylococcus* spp., and *H. influenzae* both on solid and liquid cultures (Allesen-Holm et al., 2006). Biofilm formation of many species relies on the incorporation of DNA into the biofilm matrix, as treatment with DNase is sufficient to inhibit biofilm formation (Moscoso et al., 2006; Rice et al., 2007). Additionally, the increased release of DNA during spontaneous phage lysis enhances biofilm formation of *S. pneumoniae* (Carrolo et al., 2010). Not only can eDNA serve a structural role for biofilm formation, but it also can be utilized as a nutrient source during nutrient-limiting conditions, also helping species persist. Specifically, *P. aeruginosa* encodes an extracellular deoxyribonuclease (DNase) that is capable of degrading DNA to utilize as a carbon, nitrogen, and phosphorous source (Mulcahy et al., 2010).

In addition to helping species survive in environments such as the respiratory tract, eDNA might also help organisms recover from damage caused by antibiotics or the immune system and also better adapt to an infection environment (Ibáñez de Aldecoa et al., 2017). Uptake of exogenous DNA is actually higher when bacteria live in a biofilm, rather than a planktonic state (Marks et al., 2012). The role of eDNA in DNA damage repair and gene transfer has been described for many respiratory pathogens (Ibáñez de Aldecoa et al., 2017). DNA damage resulting from insults including antibiotic treatment and the host immune system often induces the expression of many genes involved in competence. Competent organisms including *H. influenzae* and *S. pneumoniae* take up extracellular environmental DNA during this process, helping these organisms adapt by benefitting from genes that might be released from other bacterial species within a microbial community (Steinmoen et al., 2002).

### Competitive Behaviors

**Figure 2:**
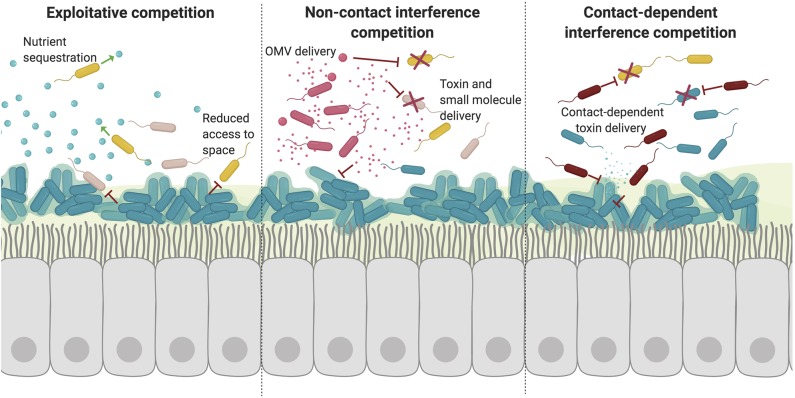
Competitive interactions disrupt microbial communities. Competitive bacterial interactions can be exploitative or interfering, which is often energetically expensive and tightly regulated. (1) Exploitative competition takes advantage of extracellular nutrients and compounds released by other species. Such competition is often for trace metals which are actively sequestered but essential for bacterial growth. Other essential resources also include access to space. Formation of recalcitrant biofilms on epithelial surfaces restricts the colonization and growth of other species. (2) Bacteria can also interfere with other species growth and signaling through the active secretion and transport of toxic molecules. There are a variety of processes used to deliver toxic molecules over a distance including direct release into the extracellular environment and packaging into vesicles. (3) Finally, specific competition with neighboring bacteria can also occur via direct contact. In this way, bacteria can specifically deliver toxins directly to other cells in close proximity through mechanisms including contact-dependent inhibition and type VI secretion.

Although many cooperative effects have been described *in vivo* and *in vitro*, many pairwise combinations of species actually result in net negative effects due to competition (Foster and Bell, 2012). Adaptation in the presence of other bacterial species is also predicted to be almost exclusively competitive to ensure the survival of a given species (Figure 2). Competition is carefully and tightly regulated in response to signals from the host and other invading microbes in order to prevent unnecessary toxicity and conserve energy (Lobato-Márquez et al., 2016).

#### Exploitative Competition

Bacteria share the need for essential nutrients and substrates in order to survive in the respiratory tract. However, not all of these molecules are released as public goods. The host often serves as a reservoir for trace minerals and nutrients, yet actively sequesters these to prevent acquisition by pathogens in a process termed “nutritional immunity (Hood and Skaar, 2012).” In order to ensure access to these restricted resources, bacteria must confiscate these nutrients for themselves or circumvent the mechanisms other microbes use to access these nutrients. These competitive behaviors not only underlie a pathogen's success in the host environment, but also have important roles in the ecology and diversity of the respiratory microbiome, impacting chronic respiratory disease (Figure 2).

#### Nutrient Access

Trace metals including iron, zinc, manganese, and copper are essential nutrients for many different bacterial enzymes (Palmer and Skaar, 2016). The ability to acquire all of these nutrients certainly drives adaptation in the host and also likely influences inter-species competition to ensure survival. Of these nutrients, the best studied example is iron. Iron is an essential cofactor for the host and microbes alike, where it is used for enzymes involved in a variety of processes including DNA replication and metabolism. Iron is sequestered by the host by iron-binding proteins and intracellular iron stores (Hood and Skaar, 2012). Many bacterial species produce secreted iron-chelating molecules, termed siderophores, that allow bacteria to access and transport ferric iron for use (Miethke and Marahiel, 2007). Some species can also utilize xenosiderophores, siderophores produced by other species. The expression of siderophores and cognate receptors is greatly increased during iron-limiting conditions, as found in the host. Although the production of siderophores might benefit neighboring species who also express the cognate receptor (xenosiderophore receptors), their production is largely competitive, as bacteria must acquire and sequester iron in order to compete for finite resources in the host. For example, when *P. aeruginosa* overproduces siderophores it inhibits the growth of *Streptococcus* spp. by restricting the amount of iron available to *Streptococcus* spp. (Scott et al., 2019). Additionally, *P. aeruginosa* encodes many outer membrane receptors to benefit from xenosiderophores in order to sequester iron for itself. In co-culture with *B. cepacia, P. aeruginosa* can detect and respond to ornibactin produced by *B. cepacia (Weaver and Kolter*, *2004**)*. By acquiring iron from other organisms, *P. aeruginosa* can easily acquire essential iron, while restricting iron acquisition from other microbes. Utilizing this iron source, rather than producing its own chelating molecules, prevents *P. aeruginosa* from expending the energy necessary to produce siderophores.

Iron can also be acquired through other mechanisms, including direct lysis of competing cells (Mashburn et al., 2005). In low iron conditions, *P. aeruginosa* can lyse *S. aureus* and *S. pneumoniae* to access their stored iron through activity of the LasA protease. The presence of *S. aureus* actually decreases *P. aeruginosa's* transcription of iron-regulated genes, as *S. aureus* alone can serve as an iron source for *P. aeruginosa* (Mashburn et al., 2005). Iron can also be acquired through heme breakdown, as 80% of host iron is present as ferriprotoporphyrin IX (heme) (Hammer and Skaar, 2011). Because heme is toxic at high concentrations, many species have developed mechanisms to process heme. *S. aureus* preferentially acquires iron from heme during iron-limiting conditions by utilizing hemolysins that function to lyse erythrocytes to gain access to hemoglobin and heme. In the presence of other *Staphylococcus* and *Corynebacterium* species, *S. aureus* has enhanced β-hemolytic activity, suggesting that *S. aureus* sequesters more iron away from other species in order to ensure its survival (Hebert and Hancock, 1985).

#### Communication Disruption

Cell to cell communication is an essential component of bacterial communities in order to modulate gene expression to best ensure survival. Although quorum sensing (QS) and inter-species communication can be used cooperatively in microbial communities, this pathway can also be exploited, allowing species to “eavesdrop” on neighboring species (Miller et al., 2018). QS can be blocked by inhibiting the production of signal, delivery of the signal, or detection of the signal. In bacteria this is often achieved by enzymatic activity, as many naturally occurring quorum quenching enzymes have been described. These enzymes are often either lactonases, which cleave the lactone ring of N-acyl homoserine lactones (AHLs) or acylases, which hydrolyze the amide bond of AHLs, both of which result in inactivation of the autoinducer. Lactonases have been described in species of *Klebsiella* and *Acinetobacter*, which are capable of attenuating the production of virulence factors, including *P. aeruginosa* elastase (Chan et al., 2011). The quorum quenching acylase PvdQ of *P. aeruginosa* is capable of inactivating the 3-oxo-C12-HSL signal in *P. aeruginosa*, and might also have some activity in decreasing the amount of C8-HSL signal in *B. cenocepacia* (Koch et al., 2014; Utari et al., 2018). Additionally, quorum quenching bacterial species capable of degrading AHLs were able to decrease *P. aeruginosa* biofilm formation and elastase production. These species also attenuated the virulence of *P. aeruginosa* and *B. cenocepacia* in a *Caenorhabditis elegans* model, supporting the role of QS in bacterial pathogenesis (Christiaen et al., 2014).

#### Growth interference

Bacteria produce an arsenal of enzymatic and non-enzymatic molecules which function to kill or interfere with microbial growth (Figure 2). Many metabolites with diverse functions from *P. aeruginosa* have antimicrobial properties, including pyocyanin and HQNO (Noto et al., 2017). *P. aeruginosa* produces four types of phenazines, redox-active pigments with broad-spectrum antibiotic properties and roles in virulence. One of these phenazines, pyocyanin, has been specifically demonstrated to reduce microbial community diversity (Norman et al., 2004). Pyocyanin inhibits the growth of many other species, including *B. cepacia*, allowing *P. aeruginosa* to outcompete *B. cepacia* in polymicrobial biofilms (Tomlin et al., 2001). However, the effects of pyocyanin also inhibit oxidative respiration in *S. aureus* (Filkins et al., 2015; Noto et al., 2017). As such, pyocyanin drives *S. aureus* into a respiration-deficient small colony variant (SCV) state and also leads to the formation of persister cells in *A. baumannii* (Bhargava et al., 2014; Noto et al., 2017). Another secondary metabolite of *P. aeruginosa*, 4-hydroxy-2-heptylquinoline N-oxide (HQNO), also functions by inhibiting respiratory electron transfer. Like pyocyanin, HQNO also inhibits the growth of *S. aureus*, while promoting the growth of SCVs, but increasing tolerance to antibiotics (Orazi and O'Toole, 2017). Additionally, HQNO also inhibits many *S. aureus* virulence factors including *agr* signaling, alpha hemolysin production, and cytochrome oxidase production (Mitchell et al., 2010).

Other species also produce a variety of secreted factors capable of restricting microbial growth. Previously, *S. pneumoniae* culture supernatants were found to inhibit the growth of *H. influenzae*, although *H. influenzae* culture supernatant had no effect on *S. pneumoniae* viability (Pericone et al., 2000). This growth inhibition is due to the production of hydrogen peroxide by *S. pneumoniae*, which is upregulated during co-culture with *H. influenzae*. Hydrogen peroxide by *S. pneumoniae* also was able to inhibit growth of *M. catarrhalis* and *Neisseria meningitidis*. *P. aeruginosa* also restricts *Streptococcus constellatus* growth and biofilm formation by the production of rhamnolipids, a type of bacterial surfactant (Price et al., 2015). Notably, tobramycin treatment of *P. aeruginosa* biofilms resulted in decreased rhamnolipid production, allowing for enhanced *S. constellatus* growth.

Many bacterial enzymes also function to inactivate toxic molecules produced by competing species. In fact, *S. aureus* produces catalase in order to decompose the hydrogen peroxide produced by *S. pneumoniae*, as previously described (Park et al., 2008). The production of catalase helps *S. aureus* survive *S. pneumoniae in vitro* and *in vivo*. However, many bacterial enzymes directly inhibit the growth of other microbes. Many of these enzymes function to degrade bacterial cell walls. The proteolytic activity of *P. aeruginosa* against *S. aureus* has been well-established (Kessler et al., 1993). The *P. aeruginosa* LasA endopeptidase cleaves peptide bonds resulting in the degradation of the *S. aureus* cell wall. Rather than being directly secreted, enzymes are also often packaged into extracellular vesicles for delivery. These outer-membrane vesicles (OMVs) are normally produced during cell growth and fuse with the Gram-negative cell membrane or adhere to Gram-positive cell walls in order to deliver cargo directly into a cell. *P. aeruginosa* OMVs seem to be particularly effective at killing other species. OMVs from *P. aeruginosa* contained peptidoglycan hydrolase, phospholipase C, protease, hemolysin, and alkaline protease (Kadurugamuwa and Beveridge, 1996; Li et al., 1998). These enzymes effectively lysed *E. coli*, other *Pseudomonas* spp., and *S. aureus*. Notably, delivery of peptidoglycan hydrolase is likely an important mechanism for cargo delivery and killing of Gram-positive species, suggesting that OMVs can target a diversity of bacterial species.

Finally, other secreted enzymes also function to inhibit bacterial virulence and persistence, rather than directly killing other species. One example is the Esp serine protease produced by *Staphylococcus epidermidis*. This protease degrades *S. aureus* biofilms and prevents further *S. aureus* biofilm formation (Vandecandelaere et al., 2014). Esp also inactivates *S. aureus* autolysins, preventing the release of DNA from *S. aureus* following lysis. As DNA is an essential component of the biofilm matrix, Esp prevents biofilm formation by restricting DNA availability. These findings complement associations documented in the nasal cavity of CRS patients, where negative correlations exist between *S. epidermidis* and *S. aureus* (Lina et al., 2003). Since *S. epidermidis* is often non-pathogenic, this represents one way that healthy microbial flora might restrict the growth and success of invading pathogens such as *S. aureus*.

#### Antibiotic Production

Many species of bacteria produce molecules specifically to kill neighboring cells and competitors. One class of antibacterial molecules that act to kill bacterial growth are bacteriocins. It is estimated that almost all species of bacteria produce at least one bacteriocin (Klaenhammer, 1988). Although various bacteriocins possess diverse antimicrobial activities, they share an extremely narrow specificity and high activity at very low concentrations. These antibacterial peptides bind to specific outer membrane receptors to be translocated through the membrane to access their specific target. Bacteriocins produced by *P. aeruginosa*, pyocins, have been well described. Over 90% of *P. aeruginosa* strains produce at least one pyocin, but often synthesize several different pyocins with DNase, tRNase, or pore-forming activities (Michel-Briand and Baysse, 2002). Although pyocins have intra-species activities, other species including *H. influenzae* are also sensitive to pyocins, likely due to similar outer membrane receptors (Filiatrault et al., 2001). Pyocin production is often in response to stress, including antibiotic treatment or immune radicals as often encountered in chronic respiratory disease. Specifically, *P. aeruginosa* pyocin production is induced following exposure to hydrogen peroxide, ciprofloxacin treatment, and anaerobic environments (Brazas and Hancock, 2005; Chang et al., 2005; Waite and Curtis, 2009). In an analysis of *P. aeruginosa* and *B. cenocepacia* CF clinical isolates, ~97% of *P. aeruginosa* strains displayed bacteriocin-like activity, while only ~16% of *B. cenocepacia* strains possessed bacteriocin-like activity. Many of the *P. aeruginosa* CF clinical isolates possessed more than one pyocin. Notably, 81% of *P. aeruginosa* isolates were able to inhibit *B. cenocepacia*, often due to RF-type pyocins (Bakkal et al., 2010).

#### Contact-Dependent Toxin Delivery

Although there are many mechanisms bacteria use to compete with surrounding organisms in the local environment, a number of more intimate interactions also exist in order to specifically compete with cells in close proximity (Figure 2). These mechanisms are contact-dependent, as they rely on physical contact to transfer toxins and other molecules from cell-to-cell. Contact dependent inhibition (CDI) is one such mechanism used to compete with nearby cells. Bacteria that possess CDI systems form a rigid extracellular filament topped with a receptor binding domain which binds to its cognate outer membrane receptors on target bacteria, secreted by a type Vb secretion system (Anderson et al., 2014). Although this mechanism has largely been shown to mediate contact with closely related cells, more evidence has been found suggesting this activity might have broader specificity than previously suggested (Virtanen et al., 2019). Interaction with other bacteria targets secretion of CdiA effector proteins with C-terminal toxin activity which often function to disrupt membranes or degrade nucleic acid in order to inhibit bacterial growth. CDI systems are characteristically involved in discriminating neighbors in a polymicrobial community as a means of self-recognition and inhibition of CDI^−^ bacteria (Anderson et al., 2014). Competition occurs on a single-cell level, as CDI^+^ cells create a single-cell-wide boundary between neighboring cells (Bottery et al., 2019). CDI systems of *Burkholderia* spp. are also responsible for excluding CDI^−^ invading cells from pre-established biofilms (Anderson et al., 2014). However spatial organization and exclusion are not the only roles of CDI systems, delivery of CDI toxins to kin cells results in increased community behaviors including increased biofilm formation (Garcia et al., 2016). In *P. aeruginosa*, CDI systems were found to have decreased virulence in acute infection models, including in *Galleria mellonella* (Melvin et al., 2017). These downstream effects likely result from post-transcriptional regulation of virulence genes.

Other contact-dependent growth inhibition systems exist, including the recently-described type VI secretion system (T6SS). The T6SS is evolutionarily related to bacteriophage proteins (Leiman et al., 2009). These contact-dependent “molecular syringes” are contractile structures that puncture cell membranes to inject toxic effectors directly into nearby cells. Although many other bacterial secretion systems function solely in the delivery of effectors to eukaryotic host cells, the T6SS is unique in that it also delivers antibacterial effectors to neighboring bacteria (Hood et al., 2010). Toxic effectors have a variety of activities resulting in cell death or growth inhibition, including nucleic acid degradation, cell wall degradation, membrane permeabilization, and transcriptional and translational inhibition (Suarez et al., 2010; Russell et al., 2011, 2013; Sana et al., 2012; Jiang et al., 2014; Tang et al., 2018). T6SS-containing species also encode cognate immunity proteins to protect themselves from intoxication by sister cells. T6SSs have been described in ~25% of Gram-negative organisms (Bingle et al., 2008). Many respiratory pathogens, including *P. aeruginosa, B. cepacia* complex, *K. pneumoniae*, and *Acinetobacter baumannii*, encode multiple non-redundant T6SSs that function to deliver an arsenal of toxins to neighboring cells (Schwarz et al., 2010; Hachani et al., 2011). T6SS toxins from *P. aeruginosa, B. cepacia* complex, *K. pneumoniae, A. baumannii*, and the opportunistic pathogen *Serratia marcescens* have been shown to be toxic to competing species including *E. coli*, however the spectrum of toxic effects on diverse classes of bacteria is still poorly described (Jiang et al., 2014; Alcoforado Diniz and Coulthurst, 2015; Hsieh et al., 2019; Spiewak et al., 2019). Gram-positive species also possess specialized secretion systems, known as the type VII secretion system (Ates et al., 2016). This system likely impacts other microbes, however, less is known about this system and how it contributes to microbial community interactions.

## Discussion/Conclusion

**Figure 3:**
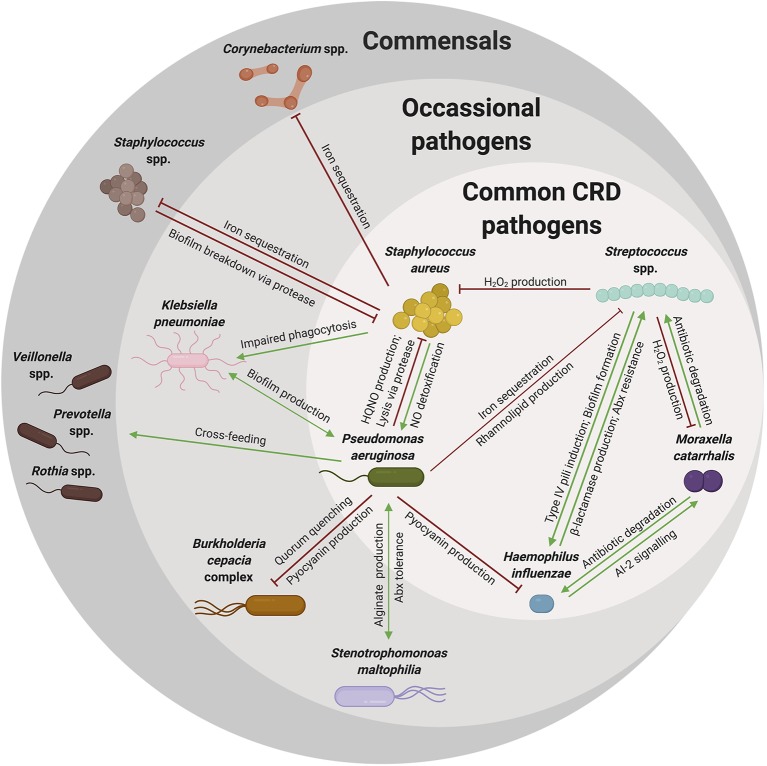
A complex web of microbial interactions shape microbial communities. Many organisms reside in the respiratory tract of young patients with respiratory disease. However, as patients age, these communities become increasingly dysbiotic, likely due to interactions among colonizing organisms and newly acquired pathogens. Many interactions have been described between commensal organisms and pathogenic species that might explain the microbial community shifts noted over a patient's lifetime. As illustrated here, many of these pathogens are shared among the various types of chronic respiratory diseases (common CRD pathogens), while other pathogens are not shared among all CRD, but rather indicated in one or few CRD (occasional pathogens), but still contribute to notable community shifts. Commensal species are also an important component of the respiratory microbiome, directly involved in competition with pathogenic species. Some of the cooperative (green arrow) and competitive (red) interactions described in this review are summarized in this web, with the arrow indicating directionality of effects. These interactions are diverse and likely complicated by respiratory symptoms and therapeutic strategies. Although complex, an understanding of these interactions can explain observed microbial shifts and may give us insight into overarching microbial community dynamics.

Microbial dysbiosis is a common marker of CRD, as those with CRD have less diversity and greater abundance of pathogenic organisms in their respiratory tract (Coburn et al., 2015; Durack et al., 2017; Copeland et al., 2018; Quaderi and Hurst, 2018). The altered community structure in the respiratory tract of CRD patients is likely shaped by respiratory symptoms and antibiotic treatment, but also is likely due to interactions within the microbial communities (Figure 3). Although many microbial interactions have been elegantly described, understanding how organisms interact in the host during CRD is still largely being elucidated. The cooperative and competitive interactions described herein may impact the resulting community structure and composition, yet there are likely many more interactions that occur that are still unknown or poorly understood. Specifically, how these interactions change in response to the environment of the host respiratory tract is still unknown. However, there are many common trends that occur across chronic respiratory diseases: (1) diseased respiratory microbiomes are dysbiotic and often result in the dominance of a single pathogenic organism over time, (2) low community diversity adversely affects lung function, and (3) disease symptoms and the respiratory environment impacts the microbiome. Although much about microbial interactions in the respiratory tract is unknown, many of the known *in vitro* microbial interactions do correlate with clinical observations. For example, *Staphylococcus* spp. and *Streptococcus* spp. have a negative correlation with one another in CRS and OM, consistent with the competitive interactions described previously. In addition to interactions with other bacteria, interactions with other organisms including viruses and fungi also impact bacterial communities. Although outside the scope of this review, viral infections are important in the pathogenesis of asthma, CF, COPD, and OM and likely impact the bacterial communities in the respiratory tract (Wedzicha, 2004; Bakaletz, 2010; Wark et al., 2012; Iikura et al., 2015). Therefore, understanding these bacterial-bacterial interactions and the polymicrobial community in the respiratory tract could be informative in understanding how to preserve microbial diversity and maintain lung function.

## Author Contributions

AW and JB wrote and edited the manuscript.

## Conflict of Interest

The authors declare that the research was conducted in the absence of any commercial or financial relationships that could be construed as a potential conflict of interest.
